# Liver transplantation for the treatment of iatrogenic bile duct injury

**DOI:** 10.1590/0100-6991e-20223436-en

**Published:** 2022-12-02

**Authors:** JOÃO OTÁVIO VARASCHIN ZENI, JULIO CEZAR UILI COELHO, CLEMENTINO ZENI, ALEXANDRE COUTINHO TEIXEIRA DE FREITAS, MARCO AURÉLIO RAEDER DA COSTA, JORGE EDUARDO FOUTO MATIAS

**Affiliations:** 1 - Universidade Federal do Paraná, Serviço de Transplante Hepático - Curitiba - PR - Brasil; 2 - Hospital Nossa Senhora das Graças, Serviço de Transplante Hepático - Curitiba - PR - Brasil

**Keywords:** Liver Transplantation, Cholecystectomy, Liver Cirrhosis, Liver Cirrhosis, Biliary, Transplante de Fígado, Colecistectomia, Cirrose Hepática, Cirrose Hepática Biliar

## Abstract

**Objective:**

to assess the outcomes of our patients who were subjected to LT for iatrogenic bile duct injury.

**Methods:**

all patients who underwent LT for treatment of complications of biliary duct injury were included in the study. Medical records and study protocols of these patients were retrospectively analyzed to determine demographic and clinical characteristics, treatment, and outcome of the patients.

**Results:**

of a total of 846 liver transplants performed, 12 (1.4%) were due to iatrogenic bile duct injury: 10 (83.3%) occurred during cholecystectomy, 1 (8.3%) following chemoembolization, and 1 (8.3%) during laparotomy to control abdominal bleeding. Cholecystectomy was performed by open access in 8 patients and by laparoscopic access in two . There were 8 female (66.7%) and 4 male (33.3%) with a mean age of 50.6 ± 13.1 years (range 23 to 70 years). All transplants were performed with livers from cadaveric donors. The mean operative time was 558.2 ± 105.2 minutes (range, 400-782 minutes). Biliary reconstruction was performed with Roux-en-Y hepaticojejunostomy in 11 patients and choledochocholedochostomy in one. Seven patients died (58.3%) and five (41.7%) were alive during a mean followed up of 100 months (range 18 to 118 months).

**Conclusion:**

liver transplantation in patients with iatrogenic bile duct injury is a complex procedure with elevated morbimortality.

## RESUMO

**Objetivo:**

avaliar os resultados dos nossos pacientes que foram submetidos a transplante hepático por lesão iatrogênica do ducto biliar.

**Métodos:**

todos os pacientes que foram submetidos a transplante hepático para tratamento de complicações da lesão do ducto biliar foram incluídos no estudo. Os prontuários e protocolos de estudo desses pacientes foram analisados retrospectivamente para determinar características demográficas e clínicas, tratamento e desfecho dos pacientes.

**Resultados:**

de um total de 846 transplantes hepáticos realizados, 12 (1,4%) foram por lesão iatrogênica de via biliar: 10 (83,3%) ocorreram durante colecistectomia, 1 (8,3%) após quimioembolização e 1 (8,3%) durante laparotomia para controle de sangramento abdominal. A colecistectomia foi realizada por via aberta em 8 pacientes e por via laparoscópica em dois. Haviam 8 mulheres (66,7%) e 4 homens (33,3%), com média de idade de 50,6 ± 13,1 anos (variação de 23 a 70 anos). Todos os transplantes foram realizados com fígados de doadores cadavéricos. O tempo operatório médio foi de 565,2 ± 106,2 minutos (variação de 400-782 minutos). A reconstrução biliar foi realizada com hepaticojejunostomia em Y de Roux em 11 pacientes e coledococoledocostomia em um. Sete pacientes morreram (58,3%) e cinco (41,7%) estavam vivos durante um seguimento médio de 100 meses (variação de 18 a 118 meses).

**Conclusão:**

o transplante hepático em pacientes com lesão iatrogênica das vias biliares é um procedimento complexo com elevada morbimortalidade.

## INTRODUCTION

Iatrogenic bile duct injury is a serious complication, with important consequences not only for patient, but also for their families, surgeons, and hospitals^1^ ^-^ ^3^. Although cholecystectomy is by far the leading cause of iatrogenic bile duct injury, other important causes are reported, including upper abdominal operations and percutaneous or endoscopic procedures. Cholecystectomy is the most common intra-abdominal surgical procedure worldwide, with an estimated incidence of bile duct injury (BDI) between 0.3-0.6%^4^ ^-^ ^7^. When the injury progresses to biliary stricture, it results in chronic exposure of the canalicular membrane to hepatotoxic bile acids. This leads to a process of ductal proliferation and portal inflammation together with fibrogenesis, known as ductal reaction, and consequent local fibrosis and cholestasis. In this context, depending on the degree of stenosis and the time of evolution, the incidence of secondary biliary cirrhosis can vary from 7% to 25% of cases^8^ ^-^ ^11^.

Most complications are initially treated with endoscopic and/or transparietohepatic dilatation of biliary stenosis^10^ ^,^ ^11^. In cases of treatment failure with these methods, surgical therapy becomes imperative. Roux-en-Y hepatojejunostomy is the most used operation, with a success rate of 79% to 93% of cases^2^ ^,^ ^4^ ^,^ ^11^. However, some patients progress to disabling complications, such as recurrent cholangitis, gallstones, secondary biliary cirrhosis, and end-stage liver disease. Liver transplantation (LT) may represent the only curative and life-saving option for the management of complicated BDI^12^ ^,^ ^15^. There are few publications on LT in patients with BDI^12^ ^-^ ^18^. In Brazil, there is only one manuscript with a small series of cases on this important subject^19^. The aim of the present study is to evaluate the results of patients who underwent LT for BDI in our hospitals.

## METHODS

We reviewed all liver transplants performed at Hospital das Clínicas, Federal University of Paraná, and Hospital Nossa Senhora das Graças, both in Curitiba, Paraná State, Brazil, from September 1991 to December 2020. We included all patients undergoing LT for the treatment of BDI complications, and retrospectively analyzed the medical records and study protocols of these patients.

We obtained and analyzed data on age, sex, indication for transplantation, blood group, cause of BDI, presence of associated vascular injury, Strasberg-Bismuth BDI classification^20^, MELD (Model for End-stage Liver Disease) score at the time of transplantation, previous percutaneous, endoscopic, and surgical treatments performed, operative findings and complications, type of transplant, transplant result, and anatomopathological result of the removed liver. Values were expressed as mean ± standard deviation (SD). This study was approved by the Ethics in Research Committee of the Hospital de Clínicas, Federal University of Paraná (Protocol approval number CAAE 40205120.2.1001.0096). Informed consent was waived due to the non-interventional and retrospective design of the study. All researchers signed a data use agreement to ensure data confidentiality and ethical use.

## RESULTS

From 846 LT performed, 12 (1.4%) were due to advanced biliary cirrhosis secondary to iatrogenic BDI. All 12 patients were referred from other hospitals.

There were eight women (66.7%) and four men (33.3%), with a mean age of 50.6 ± 13.1 years (range 23-70). Regarding blood type, seven recipients were type O, two were type B, and three were type A. The mean MELD score determined on the day of transplantation was 24.2 ± 4.34 (range 19-34) (Table 1).

**Table 1:** Demographic, clinical, treatment, and liver transplant outcome data in patients with bile duct injury.

Patient	1	2	3	4	5	6	7	8	9	10	11	12
Age on the day of the BDI	50	23	70	52	39	47	57	50	53	68	32	36
Genre	male	male	female	female	female	female	male	male	female	female	female	female
Procedure that caused the BDI	Chemo	OC	Lap	OC	OC	OC	OC	OC	OC	OC	LC	LC
Type of injury (Strasberg classification)	E3	E3	E2	E1	E2	E1	E1	E2	E2	E2	E2	E2
Number of endoscopic procedures	2	2	0	5	1	4	0	1	4	6	4	4
Number of radiological procedures	3	1	3	4	4	3	6	1	4	2	3	1
Number of surgical procedures	3	1	2	2	1	2	1	2	2	2	2	1
Time from BDI to LT (months)	60	90	78	54	93	80	96	23	54	24	120	58
MELD score on LT day	23	27	23	21	20	19	34	27	28	24	22	23
Type of biliary reconstruction in LT	HJ	CC	HJ	HJ	HJ	HJ	HJ	HJ	HJ	HJ	HJ	HJ
LT duration (min)	615	620	565	580	540	660	450	535	782	470	320	520
LT result	Death	Death	Death	Death	Alive	Alive	Death	Death	Death	Alive	Alive	Alive

BDI: bile duct injury; LT: liver transplantation; MELD: model for end-stage liver disease; Chemo: chemoembolization; Lap: laparotomy; OC: open cholecystectomy; LC: laparoscopic cholecystectomy; CC: choledocholedocostomy; HJ: Hepaticojejunostomy.

The most common cause of BDI was iatrogenic injury during cholecystectomy (n=10; 83.3%), whether by laparotomy (n=8) or laparoscopy (n=2). One patient (8.3%) had injury to the right intrahepatic bile duct after chemoembolization and one (8.3%) had injury to the common hepatic duct during laparotomy performed to control bleeding caused by a stab wound to the hepatic hilum.

The lesion was in the common hepatic duct in seven patients (58.3%), in the common bile duct in three (25%), in the right intrahepatic hepatic duct in one (8.3%), and in both right and left hepatic ducts in one (8.3%). Patients were classified according to the Strasberg classification modified by Bismuth^20^ in type E2 in seven patients (58.3%), type E1 in three (25%), and type E3 in two (16.7%). The right hepatic artery was ligated in one patient (8.3%). Portal vein injury was not recorded.

Bile duct injury was identified during cholecystectomy in six (50%) patients. Of these, four were treated with Roux-en-Y hepaticojejunostomy and later required radiological and endoscopic interventions with stent placement. The mean catheter exchange was 4.67 times (range 4-6). One patient underwent primary suture of the lesion and had to undergo five endoscopic retrograde cholangiopancreatography (ERCP) procedures, and subsequently underwent Roux-en-Y hepaticojejunostomy, which culminated in the need for three other radiological interventions with percutaneous transhepatic cholangiography (PTHC). One patient was initially treated with external drainage with a Penrose drain and was referred to our center. This patient underwent Roux-en-Y hepaticojejunostomy in one month and later also required two radiological interventions with PTHC.

In the remaining six patients (50%), the injury was diagnosed only after the procedure that caused the injury. Four patients underwent ERCP, with a mean of 4.67 (range 4-6) procedures. Subsequently, these patients required Roux-en-Y hepaticojejunostomy and other radiological and endoscopic procedures. The mean catheter exchange was 3 (range 2-4). One patient underwent a hepaticojejunostomy four days after the stab wound and subsequently required three radiological procedures with PTHC. In only one patient, who underwent four sessions of chemoembolization, surgical drainage of a bilioma and three radiological procedures with PTHC were required. All these patients developed biliary stricture and consequent secondary biliary cirrhosis with advanced liver failure, and underwent LT. The mean time from diagnosis of BDI to LT was 62.1 ± 35.4 months (range 23-120).

All transplants were performed with cadaveric donor livers. The mean operative time was 558.2 ± 105.2 minutes (range 400-782). Biliary reconstruction was performed with Roux-en-Y hepaticojejunostomy in 11 patients, and choledocholedocostomy, in one.

All patients required operative transfusion of packed red blood cells, with a mean of 6.78 ± 1.61 units (range 5-10). A mean of 5.9 ± 2.76 units (range 1-10) of plasma and 4.3 ± 3.46 units (range 0-10) of platelets were transfused.

In the first 30 days after surgery, there was a need for transfusion of 3.4 ± 2.66 (range 0-8) units of packed red blood cells, 3.6 ± 4.52 (range 0-14) units of plasma, and 2.2 ± 3.45 (range 0-10) units of platelets.

There was a biliary complication with fistula in only one patient. This patient underwent a hepaticojejunostomy that developed a fistula in the immediate postoperative period. Postoperative acute renal failure occurred in four patients.

Of the 12 patients, seven died (58.3%), three within the first 24 hours from refractory hemorrhagic shock and the other four within the first six months after transplantation from acute renal failure, gastrointestinal bleeding, and septic shock of pulmonary origin. The other five (41.7%) patients are alive, with a mean follow-up of 100 months (ranging from 18 to 118 months).

The anatomopathological examination showed hepatic cirrhosis and significant ductal dilatation. No associated carcinoma was identified in all twelve explanted livers.

## DISCUSSION

BDI is one of the most serious and feared abdominal surgical complications. It is a potentially devastating event for the patient. Complications include biliary fistula and/or stenosis, recurrent cholangitis, secondary biliary cirrhosis, and liver failure^11^ ^,^ ^21^ ^,^ ^22^. Patients with BDI also experience significant emotional and financial stress, with severe impairment of quality of life for many years or even for life 6. In addition, the rate of lawsuits is quite expressive^12^ ^,^ ^23^ ^,^ ^24^.

About 80% to 90% of BDI occur in patients undergoing cholecystectomy^13^ ^,^ ^25^. Currently, the rate of BDI is similar in patients undergoing open or laparoscopic cholecystectomy and varies between 0.1% and 0.6%^4^ ^,^ ^12^ ^,^ ^26^ ^,^ ^27^. However, laparoscopic cholecystectomy is associated with more severe injuries due to the more proximal location of the injury in the biliary tree and the frequent association with vascular injury^4^ ^,^ ^12^. Several other surgical procedures in the upper abdomen can also cause BDI, such as liver transplantation, hepatectomy, gastrectomy, lymphadenectomy, and portocaval bypass^3^ ^,^ ^4^. Biliary strictures have also been reported after radiotherapy, radioablation, chemoembolization, injection of sclerosing substances in hemorrhagic duodenal ulcer, and after blunt or penetrating trauma to the bile duct^4^ ^,^ ^21^.

Similar to other studies, the main cause of BDI in our series was cholecystectomy. Although the risk of BDI is low in a patient undergoing cholecystectomy, in general this operation is the main cause of BDI, as cholecystectomy is the most commonly performed abdominal operation worldwide^5^. Most studies, including ours, show that the rate of BDI is higher in females, as cholecystectomy is performed three to four times more commonly in this gender due to the higher prevalence of gallstones in females^5^ ^,^ ^13^.

Unlike publications from the USA and Europe, but similar to another Brazilian study, most of our patients were initially submitted to open cholecystectomy^2^ ^,^ ^7^ ^-^ ^11^ ^,^ ^13^ ^,^ ^19^. Possibly, this observation is due to the fact that most of our patients were initially operated on in small hospitals, where most cholecystectomies are performed by laparotomy.

The adequate treatment of BDI depends on the time interval from which the lesion is recognized, its extension, the patient’s clinical condition, and the availability of adequate equipment and experienced hepatobiliary specialists^4^ ^,^ ^6^ ^,^ ^13^. A multidisciplinary team, including an experienced endoscopist, interventional radiologist, and hepatobiliary surgeon, is of paramount importance to achieve the best outcome. Ideally, the patient should be referred to a center with expertise in complex hepatobiliary surgery. As most surgeons currently have more experience with laparoscopic procedures than with laparotomy ones, it may be preferable to send patients with BDI to an appropriate referral institution^6^ ^,^ ^7^.

Some factors such as delay in establishing the correct diagnosis, late referral to the tertiary center, injury at or above the right and left bile duct junction, simultaneous vascular injuries, and multiple previous surgical procedures for the treatment of BDI have a negative impact on the BDI long-term treatment outcome^4^ ^,^ ^6^ ^,^ ^13^ ^,^ ^27^. Some patients with BDI associated with severe damage to the hepatic artery and portal vein may develop acute liver failure due to massive hepatic necrosis and require emergency liver transplantation^6^ ^,^ ^12^ ^,^ ^19^.

All of our 12 patients who underwent LT for BDI had multiple sessions of endoscopic and radiological treatment. All patients also had at least one surgical procedure. Roux-en-Y hepaticojejunostomy was the most common operation.

Several authors have also demonstrated that multiple endoscopic, radiological, and surgical procedures are often necessary to treat major BDI and its complications^13^ ^,^ ^19^ ^,^ ^25^. Despite these diverse therapeutic options, some patients progress to a critical condition due to complications secondary to biliary cirrhosis and chronic liver failure. LT may be the only final treatment option for these patients.

In our series, the average time interval between BDI and LT was very long, six years. This long time possibly reflects the extended period required for the liver parenchyma to progress to advanced liver cirrhosis and failure. Multiple endoscopic, radiological, and surgical treatments employed in these patients possibly delayed the development of advanced liver failure. Chiche et al.^25^ and Silva Filho et al.^19^ also reported a long time interval between the occurrence of BDI and LT.

Although BDI is an uncommon indication for LT, this is a matter of great importance, as it often occurs in adults who were initially operated on for a benign disease. The rate of LT for BDI varies in the international literature^22^. In a National Review of the US UNOS Database, Garcia et al.^13^ reported that of the 101,238 liver transplants performed between 1994 and 2014, 61 (0.06%) were related to BDI. The rate of LT for BDI was 0.13% (30 out of 23,329 LT) in 11 French LT centers^25^. In Buenos Aires, Argentina, de Santibañes et al.^12^ described the highest rate, 2.4% (16 out of 663 LT). In the State of Ceará, Brazil, Silva Filho et al.^19^ reported a LT for BDI rate of 0.60% (10 of 1,662 LT), close to our 1.4%.

LT in patients with BDI is usually a procedure of great technical complexity and high morbidity and mortality. The presence of extensive adhesions resulting from previous surgeries to correct the BDI, combined with liver cirrhosis, portal hypertension, and biliary infection caused by resistant bacteria, make LT an enormous surgical challenge in patients with BDI^23^ ^,^ ^25^. The duration of the operation and the operative bleeding are extensive, even when LT is performed in a referral center^13^ ^,^ ^23^ ^,^ ^25^.

Some authors have shown that the operative mortality and morbidity rates of LT in patients with BDI are much higher than for other indications^19^ ^,^ ^25^. Addeo et al.^16^ reported a mortality of 61% for BDI-related LT. Our mortality rate of 58.3% was also significant. Most of our patients died of hemorrhage and acute renal failure within the first postoperative month.

The main limitations of our study are the small number of patients and the retrospective evaluation of the data. The vast majority of studies on LT in patients with BDI in isolated medical institutions include figures similar to ours, between six and 11 patients^16^ ^,^ ^18^ ^,^ ^19^. The BDI corresponds to only 0.06% to 2.4% of all LT worldwide^13^ ^,^ ^21^ ^,^ ^25^. The reduced number of BDI undergoing LT limits the possibility of carrying out studies with large numbers of patients in a single institution.

Some multicenter studies were recently published to overcome this limitation, but they lack standardized medical management^13^ ^,^ ^21^ ^,^ ^25^. BDI is a serious medical complication that must be avoided. Therefore, prospective studies are impossible to be carried out. In our series, this is minimized because all medical and surgical procedures were coordinated and supervised by the same transplant team and data were retrieved from electronic medical records and study protocols.

There is a lack of studies on LT in patients with BDI in South America^12^ ^,^ ^21^. There is only one Brazilian manuscript^19^. As results vary across the world regions, our study could be a valuable contribution to this important issue.

## CONCLUSION

Liver Transplant may be the only life-saving treatment for BDI patients with advanced liver failure. LT in patients with BDI is a complex procedure, with high morbidity and mortality.
